# Apolipoprotein M—A Marker or an Active Player in Type II Diabetes?

**DOI:** 10.3389/fendo.2021.665393

**Published:** 2021-05-21

**Authors:** Christina Christoffersen

**Affiliations:** ^1^ Department of Clinical Biochemistry, Rigshospitalet, Copenhagen, Denmark; ^2^ Department of Biomedical Sciences, University of Copenhagen, Copenhagen, Denmark

**Keywords:** apolipoprotein M, sphingosine-1-phosphate, obesity, diabetes, insulin resistance

## Abstract

Apolipoprotein M (apoM) is a member of the lipocalin superfamily and an important carrier of the small bioactive lipid sphingosine-1-phosphate (S1P). The apoM/S1P complex is attached to all lipoproteins, but exhibits a significant preference for high-density lipoproteins. Although apoM, S1P, and the apoM/S1P complex have been discovered more than a decade earlier, the overall function of the apoM/S1P complex remains controversial. Evidence suggests that the complex plays a role in inflammation and cholesterol metabolism and is important for maintaining a healthy endothelial barrier, regulating the turnover of triglycerides from lipoproteins, and reducing cholesterol accumulation in vessel walls. Recent studies have also addressed the role of apoM and S1P in the development of diabetes and obesity. However, limited evidence is available, and the data published so far deviates. This review discusses the specific elements indicative of the protective or harmful effects of apoM, S1P, and the apoM/S1P complex on type 2 diabetes development. Since drugs targeting the S1P system and its receptors are available and could be potentially used for treating diabetes, this research topic is a pertinent one.

## Introduction

Apolipoprotein M (apoM) was discovered in 1999 (1) and has since been associated with various conditions, such as atherosclerosis, cardiovascular disease (2), dyslipidemia (3), diabetes (4, 5), inflammation, and sepsis (6). ApoM is expressed in the liver and kidney, and to a minor extent, in adipose tissue (2, 7). While circulating apoM is primarily bound to and carried by high-density lipoproteins (HDLs), it can also bind to other lipoprotein subtypes, such as low-density lipoproteins (LDLs), very-low-density lipoproteins (VLDLs), and chylomicrons (1, 8). Most apolipoproteins are associated with a single class of lipoproteins; however, the promiscuous nature of apoM may lead to complexities related to its biological functions. For example, apoM-containing HDL was shown to reduce cholesterol deposition in the walls of blood vessels, thereby reducing atherosclerosis in animals (2, 8, 9). In contrast, apoM-containing LDL increased the circulation time of LDL particles and potentially increased the risk of deposition of such particles in the vessel walls (3, 10). Additionally, apoM can switch between lipoprotein subtypes depending on the relative concentration of each subtype (10). The marked difference in the enzyme and lipoprotein distribution in humans and mice further complicates this phenomenon. Therefore, it remains unclear whether apoM plays a positive or negative role in lipid metabolism, and details of the biological role of apoM are yet to be uncovered. The onset of diabetes makes it more challenging to identify the precise role of apoM owing to its contribution in dyslipidemia, insulin resistance, and inflammation, which potentially affects the plasma levels of apoM. Importantly, these biological conditions may also be modified by apoM per se and will be discussed further in this review.

**Figure 1:**
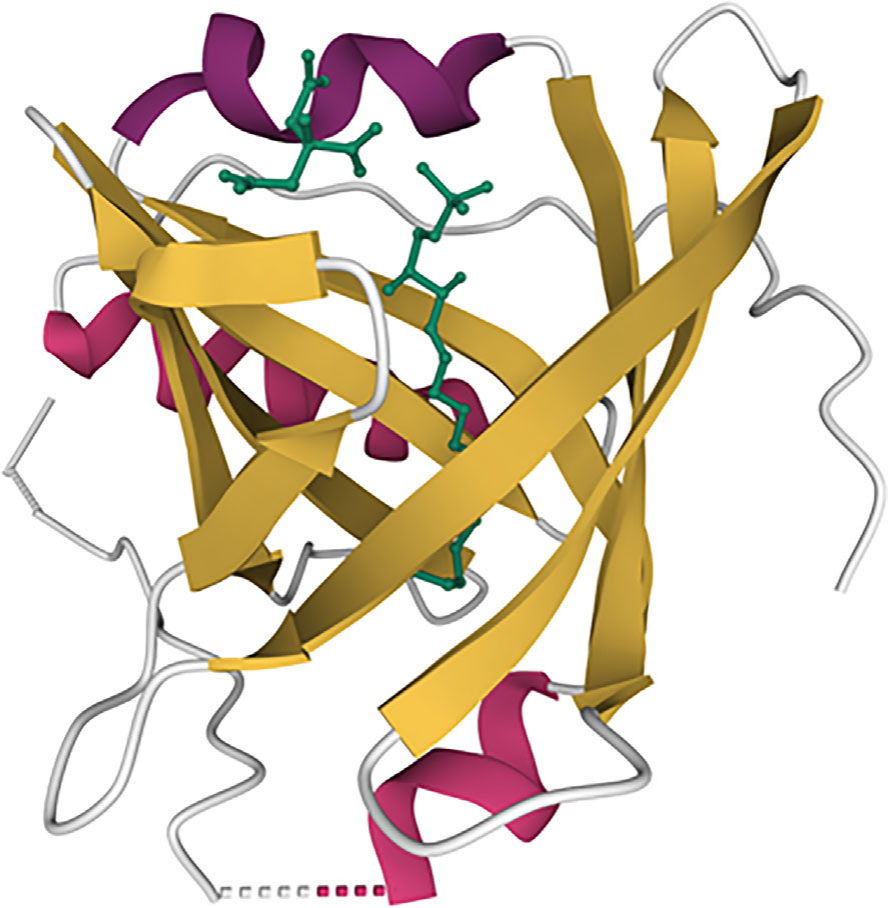
Illustration of the apoM structure with S1P. Beta-sheets are represented by yellow lines and alpha helix are represented by purple colors. The S1P molecule is represented by the green line. The model is based on the RCSB protein data bank ID 2YG2 (11).

**Figure 2:**
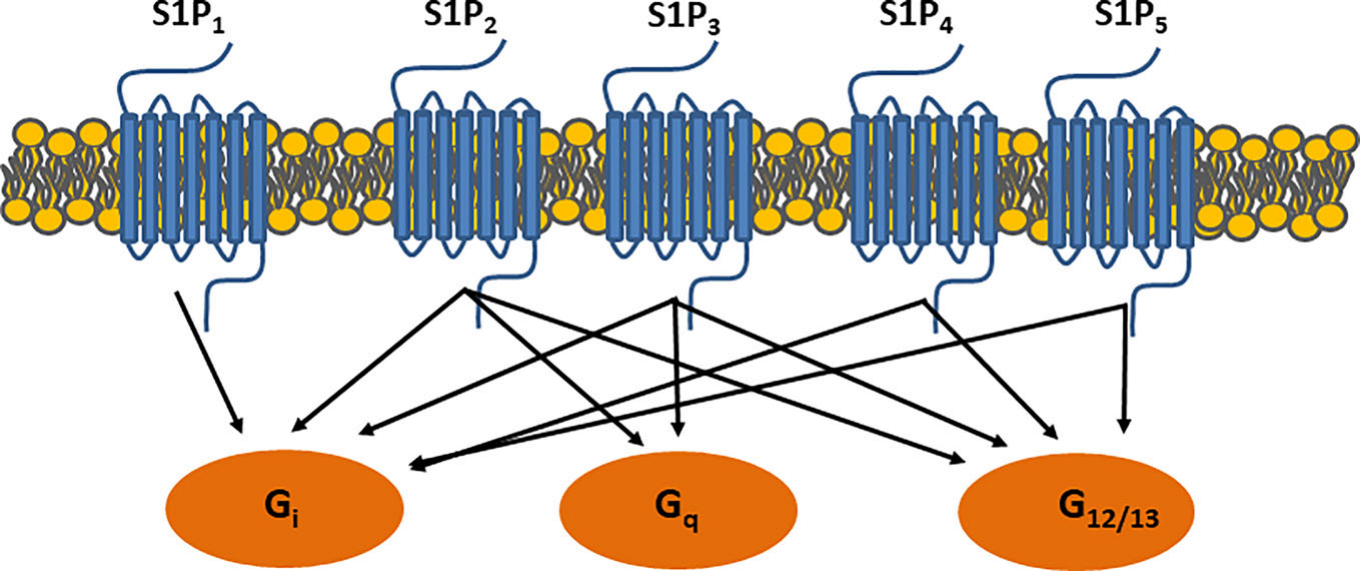
S1P receptor signaling. Binding S1P-receptors initiates signaling through G-protein coupled receptors, which further activate small GTPase binding proteins i.e. Ras, Rac, and Rho.

ApoM is characterized by its ability to structurally form a hydrophobic binding pocket as a member of the lipocalin superfamily (11, 12) (**Figure 1**). In this central pocket, apoM can bind the small bioactive lipid, sphingosine-1-phosphate (S1P). ApoM was found to be an important chaperone for S1P and a participant in the interaction with the S1P-receptor family (11). Five G-protein-coupled receptors (S1P1–5) have been identified to date. S1P1–3 are expressed ubiquitously, whereas the expression of S1P4 is restricted to the lungs and lymphoid organs, and that of S1P5 to the brain, skin, spleen, and leukocytes (13). When S1P binds to S1P receptors, signaling is initiated by the phosphorylation of the G-proteins associated with each S1P receptor, which induces the internalization of the S1P-S1P-receptor complex (**Figure 2**). The binding of S1P to S1P1 activates Gi (14), binding to S1P2–3 activates Gi, Gq, or G12/13 (15) and binding to S1P4–5 activates Gi or G12/13 (16). In general, S1P1 activation is counteracted by S1P2 activation and supported by S1P3. S1P is associated with the regulation of angiogenesis (17, 18), vascular maturation during development, promotion of endothelial cell migration (19), regulation of lymphocyte trafficking (20), and enhancement of endothelial barrier function (21, 22) through these signaling cascades. S1P is produced from ceramide and sphingosine *via* sphingosine-1-kinase and sphingosine-2-kinase, which are widely distributed. S1P can be converted to sphingosine by S1P phosphatase 1 and 2 and degraded by S1P lyase.

The S1P signaling cascade is potent due to the various biological outcomes mentioned above and has introduced a novel dimension to apoM biology and complexity to the functionality of apoM-containing lipoproteins. This review focuses on the roles of apoM and S1P in diabetes and our learnings from human and animal studies. The role of the apoM/S1P complex as a biomarker, the effect of diabetes on the plasma apoM/S1P levels, and the potential role of apoM/S1P in the development of diabetes have been discussed.

## Can Plasma apoM Levels Contribute to the Subclassification of Diabetes?

To improve treatment and clinical guidelines for patients with diabetes, efforts have been made to subcategorize classical type I (T1D) and II (T2D) diabetes. Among these, maturity onset diabetes of the young (MODY) is difficult to distinguish from other types of diabetes unless a specific family of genetic variants is identified. Some of the genes related to MODY are members of the hepatonuclear factor (HNF) family. An insightful study conducted in both mice and humans was the first to suggest that HNF1α is an important regulator of apoM transcription (4). HNF1α-KO mice did not exhibit apoM expression in the liver and kidneys. The serum apoM levels were also reduced by 50% in HNF1α-KO mice compared to that in wild-type animals. In parallel, the study also identified that patients with mutations in HNF1α (MODY3, n=9) showed reduced serum apoM levels compared to controls (n=9) (P<0.02) and patients with mutations in HNF4α (MODY1, n=9). This study suggested that plasma apoM is a useful serum marker for the identification of patients with MODY3. Another study was performed on 71 carriers of MODY3 and 85 family controls (23). Only female carriers of MODY3 showed a 10% reduction in the plasma apoM levels. The reduction was maintained after adjusting for diabetes. The second cohort in the same study included 24 patients with MODY3, 11 patients with MODY1, 18 patients with T2D, and 19 non-diabetic controls. No differences were observed in the apoM levels across the different groups. Plasma apoM levels were measured using two different types of ELISA; however, the levels could not be used in isolation to explain the discrepancy between the conclusions. A follow-up study was conducted in a larger cohort (24). The apoM levels were measured in patients with MODY3 (n=69), T1D (n=50), T2D (n=120), and healthy controls (n=100). Again, the serum apoM levels were significantly lower in patients with MODY3 than in controls and patients with T1D. There was no significant difference in the apoM levels between patients with MODY3 and T2D.

The findings from the above studies indicate that the plasma apoM levels may be reduced in patients with MODY3 than in controls and patients with T1D; however, plasma apoM levels are not useful for distinguishing patients with MODY3 from those with T2D. Since patients with MODY have a mixed phenotype, with plasma profiles similar to those of T2D but onset at a young age as in T1D, the plasma apoM levels may be useful for partial distinction between patients with MODY3 and T1D. Further experiments, including measurement of plasma S1P levels, are needed for clarification.

## How Are Plasma apoM and S1P Associated With Lipids in Patients With Diabetes?

Diabetes is often associated with dyslipidemia. A typical lipid profile from individuals with T2D shows increased plasma triglyceride levels and reduced HDL-cholesterol levels. In addition, a preference for small atherogenic LDL particles despite normal LDL-cholesterol levels is often reported. Under normal physiological conditions, the plasma apoM and S1P levels are positively associated with the HDL- and LDL-cholesterol content in humans (25). ApoM may even exchange lipoproteins depending on the availability of the lipoprotein subtypes (10).

Plasma apoM levels were 9% lower in patients with T2D (n=78) than in controls (n=89, P=0.01) (26). The difference in apoM levels was largely attributable to diabetes-associated obesity. The apoM levels were also positively correlated with both HDL- (r=0.16; P=0.04) and LDL-cholesterol content (r=0.28; P=0.0003). The plasma apoM levels can also predict pre-b-HDL levels (r=0.16; P=0.04) and pre-b-HDL formation (r=0.19; P=0.02), independent of positive relationships with apoA-I and HDL-cholesterol. This suggests that apoM may play a role in early HDL remodeling in humans. Since apoM is associated with both HDL and LDL particles, studies have addressed the effect of LDL-reducing statin treatment on apoM levels. A study on 14 patients with T2D was included in a crossover study on treatment with statins, fibrates, and a combination of both (27). Statins reduced the plasma apoM levels by 9%. It remains unknown if this minor reduction is attributable to the absence of a normal insulin response in patients with T2D or if it is applicable to non-diabetic patients. Another interesting contributor in LDL-metabolism is proprotein convertase subtilisin/kexin type 9 (PSCK9), which controls the number of LDL receptors on the cell surface. In a study on non-diabetic individuals (n=79), overweight individuals (n= 32), and individuals with obesity (n= 10), the plasma apoM levels were inversely correlated with BMI, and the PCSK9 levels were positively correlated with insulin levels. However, despite the relationship among apoM, PCSK9, and apoB-containing lipoproteins such as LDL, the correlation between the apoM and PCSK9 levels was not significant in the entire cohort (P=0.19). The apoM levels correlated positively with PCSK9 levels in lean individuals (r= 0.337, P= 0.041) after adjustment for BMI and apoB, but not in individuals who were overweight or had obesity, suggesting that LDL-associated apoM may be differently processed depending on metabolic health.

Interestingly, S1P and apoM may also be affected in contradictory manners in individuals with obesity. The circulating S1P levels were elevated in both obese mouse models and in humans with obesity than in lean healthy controls (28). Furthermore, in humans, the plasma S1P levels were positively correlated with the total body fat percentage, BMI, fasting insulin, total cholesterol, and LDL-cholesterol. The reason for this discrepancy between the apoM and S1P levels remains unknown and requires further investigation.

Lipid metabolism is highly dependent on the responses to glucose and insulin not only in the liver but also in muscles and adipose tissues. Recently, apoM was identified as a novel adipokine (7) in a study in which plasma samples and adipose tissues from 485 individuals were tested. *APOM* is expressed in human subcutaneous and visceral adipose tissues. ApoM was also secreted from adipocytes into circulation, as measured using the arterio-venous difference from adipose tissue. *APOM* expression was inversely correlated with adipocyte size in adipose tissue, lower in obese than in lean individuals, and lower in patients with metabolic syndrome and T2D. However, regardless of the fat depot, there was a positive relationship between adipose *APOM* expression and systemic insulin sensitivity, independent of the fat mass and plasma HDL-cholesterol content. Lastly, in individuals with obesity, calorie restriction promoted adipose *APOM* expression and secretion from adipose tissue explants. It will be interesting to note whether further investigation can reveal the role of apoM and S1P in adipocyte biology and function.

The metabolic changes induced by the life-long derangement of insulin, glucose, and lipid levels can lead to an increased risk of cardiovascular disease in patients with T2D. It was evaluated whether changes in the plasma apoM or S1P levels were associated with prevalent subclinical atherosclerosis in 545 individuals from the African American Diabetes Heart Study (29). The plasma S1P and apoM levels were not associated with the coronary artery calcium levels, a marker of subclinical atherosclerosis. After 64 months of follow-up, higher levels of plasma S1P (odds ratio [OR]=0.14, P=0.01) and plasma apoM (OR=0.10, P=0.02) were found to be associated with lower mortality after adjusting for age, sex, statin use, calcium levels, kidney function, and albuminuria. The association of apoM with mortality has been confirmed previously in a cohort of patients with heart failure (30). Even though the underlying mechanism remains unknown, the effect seems to occur independently of diabetes.

## Can apoM/S1P Contribute tothe Diagnosis of Diabetic Nephropathy (DN)?

ApoM is primarily expressed in the liver, but also to a large extent in the cells of the renal proximal tubule. Diabetes is known to affect kidney function and biology, thereby inducing nephropathy. The role of apoM and S1P in the development of DN has only been addressed in a limited number of studies, but with various outcomes dependent on study design, size, and primary aim. In a study on T2D, individuals were segregated based on the degree of albuminuria (31). The plasma S1P levels declined with kidney function, as explained by the loss of albumin in urine. The study did not report any effects on the apoM levels. The urine S1P levels were not measured, and the study did not include a healthy control group. A second study included 96 patients with DN, 100 age- and sex-matched diabetic patients without DN, and 110 healthy controls. All patients with T2D were divided into three groups according to urinary albumin excretion. Patients with DN had higher plasma apoM concentrations than those without DN (P<0.05). The areas under the curve in a receiver-operating characteristic curve analysis for apoM showed that the plasma apoM levels were not a predictor of DN in healthy individuals. Surprisingly, the apoM levels were higher in patients with nephropathy and T2D. A study conducted in patients with chronic kidney disease (CKD), of whom 30% also suffered from T2D, showed a reduction in plasma apoM levels with severity of CKD in a cohort of more than 400 patients and 35 controls (32). In another study, HDL was isolated from 20 controls and patients with CKD (33). The apoM contents in HDL and plasma albumin were found to be low, whereas the HDL-S1P levels were high. Further studies are needed to understand whether apoM and/or S1P play a role in the development of DN, but also whether the complex can predict the risk of future events or deteriorating kidney function.

## Does Experimental Diabetes Affect Plasma apoM and S1P Levels?

Multiple animal models of obesity and diabetes are commercially available, and a large group of studies addressing apoM/S1P biology and its potential role in diabetes have been performed using experimental diabetic models. To interpret the outcomes of such studies, a basic understanding of the changes in plasma apoM/S1P levels during experimental diabetes and obesity is necessary. In the following section, a systematic overview of the common animal models of diabetes and the effects on plasma apoM and S1P levels has been provided (**Table 1**).

**Table 1:** Overview of the experimental animal models and the effect on either plasma apoM and S1P or effects on insulin and glucose metabolism.

Model	Diet	S1P-modulating treatment	Plasma apoM	Plasma S1P	Blood glucose	Glucose tolerance	Insulin tolerance	Reference
Wild type Rats	Glucose		Reduced					(34)
Ob/Ob	Chow		Reduced	Increased	Increased	Intolerant	Insulin resistance	(5, 28, 35)
Ob/ob	Chow	JTE-013	Unknown	Unknown	Unknown	Improved	Improved	(36)
Db/Db	Chow		Reduced	Increased	Increased	Intolerant	Insulin resistance	(28, 35)
NOD	Chow			Local increase	Increased			(37)
NOD mice	Chow	Ponesimod			Reduced	Improved	Improved	(38)
NOD.Scid mice	Chow	FTY720			Reduced	Improved	Improve	(39)
STZ-treated C57B6 mice	Chow		Increased	Increased	Increased			(40)
STZ-treated C57B6 mice	Chow	FTY720			Reduced	Improved	Improved	(41)
STZ-treated Wistar rat	Chow	FTY720			No effect	No effect	No effect	(42)
Alloxan treated NMRI mice	Chow		Reduced		Increased			(43)
C57B6 + apoM-adenovirus	HFD		Increased	Increased	Reduced	Improved	Not affected	(44)
C57B6 + apoM-adenovirus	HFD	VPC23019	Increased	Increased	Lost effect	Lost effect	Lost effect	(44, 45)
C57B6 + apoM-adenovirus	HFD		Increased	Increased	Reduced		Improved	(45)
C57B6 + apoM-adenovirus	HFD	JTE-013	Increased	Increased	Lost effect		Lost effect	(45)
ApoM-KO	HFD		Absent	Reduced	Increased	Intolerant	Insulin resistance	(45)
ApoM-KO	HFD		Absent	Reduced	Reduced	Improved		(46)
S1P2-KO	HFD		Unknown	Unknown	Reduced	Improved	Improved	(36)

C57B6, wild type mice; FTY720, S1P-receptor antagonist; HFD, high fat diet; JTE-013, S1P2 antagonist; NOD, Non-obese diabetes; STZ, streptozotocin; VPC23019, S1P1 and S1P3 antagonist.

### Animal Models of Obesity

Ob/ob or db/db mice with leptin or leptin receptor deficiency are commonly used as models of obesity and concomitant hyperinsulinemia, respectively. These models lack control of food consumption, which leads to an increase in the body weight and plasma insulin, with the potential risk of insulin resistance development, resembling obesity in humans. Both ob/ob and db/db mice showed a significant reduction in plasma apoM levels (approximately 30%–50%) (35). The reduction was related to a decrease in liver and kidney apoM mRNA expression and could be reversed by leptin administration (0.5 or 1.5 µg/g body weight). In contrast, the plasma S1P levels were elevated in obese mice compared to those in lean controls (28). The increase in plasma S1P levels was more than 100% in animals administered a high-fat diet (HFD), and approximately 50% in ob/ob mice, compared to that in chow-fed or lean control animals. The reasons for the inconsistency between the plasma apoM and S1P levels in obesity and HFD administration, as illustrated in the two studies discussed above, are unknown; however, the inconsistency has also been reported in human studies. It was expected that a reduction in plasma apoM levels would be accompanied with a similar reduction in plasma S1P levels. In wild-type animals fed a chow diet and not exposed to metabolic stress, approximately one-third of the apoM-particles contained and transported S1P, corresponding to approximately 70% of the total plasma S1P content (11). The remaining plasma S1P was bound to albumin. Furthermore, under normal physiological conditions, the plasma apoM and S1P levels were positively correlated. However, it remains unknown whether the binding potential of apoM changes in individuals with obesity, thereby counterintuitively increasing the plasma S1P levels or altering the distribution of S1P between apoM and albumin. Notably, a small proportion of S1P molecules may also bind to apoA-IV when the apoM content is depleted (47). However, it remains unknown if S1P bound to apoA-IV plays a biological role in diabetes comparable to that of S1P bound to apoM. ApoM metabolism may also be directly regulated by insulin or glucose. Hence, rats injected with a 25% glucose solution (2.5 mL/h for 6 h) showed a significant reduction in both serum apoM content and hepatic apoM mRNA levels (34). Meanwhile, the insulin level only increased moderately, indicating that the low apoM expression in these diabetic models may be ascribed to hyperglycemia rather than to insulin resistance. However, this is debatable because insulin can affect the transcription factor forkhead box A2 (FOXA2) (5), and thereby repress apoM expression. Therefore, ob/ob mice exhibited a sudden decrease in apoM expression owing to the inactivation of FOXA2 in the hyperinsulinemic state. This was the first study to identify a biological link between insulin levels and apoM transcriptional regulation in obesity.

The increase in plasma S1P levels in hyperinsulinemia may be linked to a change in the expression of multiple enzymes involved in S1P production (sphingosine kinase 1 and 2) or degradation (S1P lyase or S1P phosphatases 1 and 2); however, the precise mechanism underlying this remains unknown.

### Non-Obese Models of Diabetes

Non-obese diabetic (NOD) mice harboring polygenetic alterations that induce the risk of diabetes are another type of genetic diabetes model. NOD mice develop insulitis owing to leukocyte infiltration in the pancreatic islets. S1P1 expression was reduced in thymocytes (CD8+ and CD4+ cells) in this model compared to that in controls (37). A decrease in S1P lyase levels was also observed in NOD thymocytes, which could potentially lead to a higher local S1P level, thereby inducing S1P1 internalization from the cell surface. Therefore, the modulation of S1P1 expression and S1P/S1P1 interactions in NOD mouse thymocytes may be part of the T-cell migratory disorder observed in T1D pathogenesis. It remains unknown if apoM acts as an S1P carrier in this state, and reports on the measurement of plasma or thymic apoM levels in such animals are yet to be published.

### Models With Chemically Induced Diabetes

Streptozotocin (STZ) is commonly used for developing models of diabetes. Depending on the administration protocol and combination with diets, STZ generally induces the partial destruction of insulin-producing pancreatic beta cells. The plasma S1P and apoM levels in STZ-treated wild-type B6 mice were approximately 30% higher than those in control mice (40). A high dose of insulin (80 IU/kg body weight) decreased both plasma S1P and apoM levels. A second study observed an increase in S1P2 expression in the renal cortex and glomeruli in STZ-treated rats (48). These rats showed a significant increase in their blood glucose levels (above 300 mg/dL). The S1P2 receptor generally counteracts the effect of S1P1 owing to the activation of different G-proteins and downstream responses (**Figure 2**); therefore, an increase in S1P2 levels may increase the permeability of the glomeruli and loss of kidney function. Even though the animals were tested for 52 weeks, no signs of nephropathy were observed. However, further experiments are required to evaluate this.

Lastly, the administration of the toxic glucose analogue alloxan (120 mg/kg) in Naval Medical Research Institute outbred mice (wild-type mice used for toxicology experiments) damaged the insulin-producing pancreatic beta cells, increased the glucose levels, and reduced the insulin levels (43). Concurrently, these mice exhibited a 70% reduction in the plasma apoM levels. Compared to controls, alloxan-treated mice exhibited a reduction in apoM mRNA and gene expression in both liver and kidney tissues. The daily administration of 5 IU/kg insulin increased the plasma apoM levels. This supports the hypothesis that insulin is an important regulator of plasma apoM levels and needs to be considered when diabetic models are discussed in future studies. The S1P levels were not measured in this study and could be an important mediator of the experimental outcome.

Collectively, these experimental models can be used to monitor changes in the plasma apoM and S1P levels under various conditions depending on the choice of experimental protocol and purpose. This also highlights the need for a better understanding of animal models of diabetes before their use in studies related to the development of obesity and diabetes. The investigation of endothelial function or dyslipidemia in relation to obesity is a potential challenge. Obesity and hyperinsulinemia are accompanied by reduced apoM levels and increased plasma S1P levels. ApoM, S1P, and the apoM/S1P complex exert independent and combined effects on lipoprotein metabolism, endothelial function, and inflammation, among others. The contribution of each component in diabetes has been explored, and will be discussed further in the subsequent sections.

## Do apoM and S1P Affect the Development of Diabetes?

These findings highlight the potential effects of metabolic changes occurring during obesity and T2D on the plasma apoM/S1P content. The following section will focus on the role of apoM/S1P in the development of experimental diabetes. Since apoM is associated with changes in lipid metabolism and cardiovascular diseases and S1P is important for inflammation, lymphocyte migration, endothelial integrity, and angiogenesis, several experimental studies have attempted to explore whether apoM and/or S1P contribute to the development of diabetes (e.g., by inducing glucose or insulin resistance) and the associated complications (e.g., cardiovascular disease, neuropathy, retinopathy, and nephropathy).

### Glucose and Insulin Response

C57BL/6 wild-type HFD-fed mice treated with an adenovirus inducing the overexpression of hepatic apoM showed lower blood glucose levels compared to mice treated with control virus (44). While an insulin tolerance test showed that insulin sensitivity was not significantly affected, a glucose tolerance test showed that apoM-overexpressing mice exhibited improved glucose tolerance. This was likely attributable to enhanced insulin secretion, a phenomenon that was reversed by treatment with an S1P1 and S1P3 antagonist (VPC 23019). Some of the underlying mechanisms can be ascribed to the apoM-induced protection against S1P degradation and an increase in *Pdx1* expression (thereby reducing the endoplasmic reticulum stress) (44). A second study also supported these observations (45), and showed that ApoM-KO mice fed HFD were glucose intolerant and exhibited worsening insulin resistance, whereas mice with apoM overexpression showed improvement of insulin resistance (45). The potential mechanisms were hypothesized to include the activation of the insulin signaling pathways, such as the AKT and AMPK pathways, and improvement of mitochondrial functions through the upregulation of hepatic SIRT1 levels. These actions of apoM/S1P appear to be mediated by the activation of S1P1 and/or S1P3. To explore the pathways underlying glucose metabolism, microarray RNA analysis and analysis of metabolites (e.g., glucose, fructose, maltose, and related metabolites) in liver tissue samples obtained from ApoM-KO and wild-type HFD-fed mice were performed (49). The majority of hepatic glucose metabolites were present at lower concentrations in the apoM-KO mice than in the wild-type mice, and genes associated with hepatic glucose metabolism were downregulated. The study also showed that the level of AKT phosphorylation in response to insulin stimulation in the muscles and adipose tissues was significantly lower in apoM-KO mice than in wild-type mice.

In contrast to the above studies that were performed in apoM-KO mice using the Crispr/Cas9 technology (removal of nucleotide no. 318–328 in the murine apoM transcript), another study conducted using an alternative apoM-KO strain (replacement of 39 base pairs in exon 2 with a NEO-cassette) showed contradictory results (46). ApoM deficiency was associated with an increased brown adipose tissue content, acceleration of postprandial triglyceride clearance, and protection against diet-induced obesity. Moreover, the data suggested that apoM-deficient mice showed an S1P-dependent phenotype reflecting diminished S1P1 stimulation. The reason underlying the contradictory results is unknown. As reported, the studies were conducted in animals with a B6 background and evaluated approximately 10 weeks after the commencement of HFD, and similar plasma S1P levels were reported at baseline. The potential confounder is that neither study reported the S1P level after HFD was administered. As discussed earlier in this review, the consumption of an HFD can increase the S1P levels, whereas in apoM-expressing models, HFD reduce apoM levels *via* the negative regulation of hyperinsulinemia. However, this information was not included in the above studies and must be considered in future studies to clarify the role of apoM/S1P in obesity and the development of insulin resistance.

The S1P1 receptor has gained interest owing to its effect on lymphocyte migration and endothelial barrier function. However, the S1P2 receptor is a counteracting partner of S1P1. A study conducted on S1P2-KO mice investigated the role of this receptor in glucose metabolism. HFD-fed S1P2-KO mice showed reduced white adipose tissue, but the differences became negligible after 4 weeks of HFD (36). However, compared to wild-type mice, HFD-fed S1P2-KO mice showed improved glucose and insulin tolerance. The administration of JTE-013 (S1P2 antagonist) to ob/ob mice also improved glucose tolerance and insulin sensitivity. Hence, S1P2 blockade may be a novel therapeutic strategy for obesity and T2D. The phenotype observed in S1P2-KO mice or JTE-013-treated animals aligned with that of apoM-KO mice in certain studies (46). It remains unknown whether apoM-bound S1P prefers specific S1P-receptors; however, it could be important to explore if such targets should be developed to improve the treatment of patients.

### Immune Response and Diabetes-Induced Complications

Fingolimod (FTY720) is commonly used for treating multiple sclerosis (50). The compound is a functional S1P antagonist that binds to four of the five S1P receptors, with the highest affinity for S1P1. After binding to the receptor, it mediates receptor internalization and degradation. In multiple sclerosis, the major role played by FTY720 (or later generations of the drug) is suppression of the immune response by reducing lymphocyte migration (50). A similar effect could be advantageous for avoiding the migration of inflammatory cells into and destruction of insulin-producing beta cells in the pancreas or organs (e.g., retina, kidney, and arterial wall) exhibiting associated immune-mediated complications (such as retinopathy, nephropathy, and cardiovascular diseases). These mechanisms have been explored in several studies. STZ-treated Wistar rats with a blood glucose concentration greater than 16.7 mM were administered FTY720 daily for 12 weeks (42). FTY720 treatment did not reverse the diabetic phenotype induced by STZ treatment. In other models of diabetes, the inhibition of the S1P-mediated immune response may delay or inhibit diabetes. For example, NOD.Scid mice (a model lacking mature B and T cells and resistance to insulitis under normal conditions) were transplanted with splenocytes (including T-cells) from diabetic NOD mice (39). The transplantation model developed diabetes, which could be prevented by administering FTY720 before the transfection of T-cells (39). If FTY720 was administered after the transplantation, the progression of diabetes would be delayed, but not prevented, since the drug would be unable to completely suppress memory and effector T-cells in circulation. Ponesimod, a second-generation drug with a selective S1P1 receptor profile, has also been tested in a NOD animal model (38). Ponesimod was administered orally to NOD mice starting at 6, 10, 13, and 16 weeks of age and continuing up to 35 weeks of age or to NOD mice with recent onset diabetes. The peripheral blood and spleen B and T cell counts were significantly reduced after ponesimod administration. Chronic ponesimod treatment efficiently prevented autoimmune diabetes in 6-, 10-, and 16-week-old pre-diabetic NOD mice. However, treatment withdrawal led to synchronized disease relapse. Collectively, these studies suggested that the suppression of the immune response *via* the S1P-S1P1 axis can potentially delay the onset of diabetes.

The development of cardiac fibrosis is not uncommon in diabetes; however, the appropriate treatment procedure is yet to be established. Interestingly, treatment with FTY720 reduced T-cell infiltration and the extent of cardiac fibrosis in STZ-treated animals (41). FTY720 exerted no protective effect in models without T-cells (such as Rag1-KO mice); rather, it accelerated fibrosis in these models. FTY720 treatment also normalized the hyperglycemia-induced plasma S1P levels and S1P1 expression in cardiomyocytes. Furthermore, animals with conditional T-cell S1P1 knockout (TS1P1KO) developed STZ-induced diabetes (51). TS1P1KO mice exhibited a sustained deficiency of both CD4+ and CD8+ T cells in blood. Meanwhile, compared to wild-type mice, vehicle-treated TS1P1KO control mice showed an altered phenotype characterized by increased myocardial fibrosis and reduced cardiac contractility. Under diabetic stress, TS1P1KO mice exhibited improved cardiac function and lower cardiac fibrosis compared to wild-type diabetic mice. Therefore, T-cell S1P1 activation exerts antifibrotic effects in normoglycemia, but exacerbates fibrosis in hyperglycemia.

STZ treatment can also induce end-stage DN with albuminuria. Treatment with FTY720 or SEW2871 (an S1P1 agonist) attenuated these effects (52). While FTY720 induced lymphopenia, SEW2871 reduced albuminuria in Rag1-KO mice (lacking T-cells), suggesting that the reduction was not associated with lymphocyte infiltration. These beneficial effects could be explained by the S1P1-induced protection of podocytes.

Lastly, retinopathy is another complication associated with diabetes. Interestingly, after FTY720 treatment, STZ-treated rats developed lymphopenia, with reduced levels of inflammatory cytokines compared to that in controls. The levels of cellular adhesion molecules, such as VCAM and ICAM, and the permeability of the retinal barrier, were also reduced in retinal biopsies. Therefore, this study suggested a potential treatment for diabetes-induced retinopathy using FTY720 (42).

## Can Genetic Variants in the *APOM* Transcript Predict Risk of Diabetes in Humans?

Based on the observation that changes in signaling *via* the S1P-receptors in animal models can delay the development of diabetes, and that plasma apoM levels may be used as a subclassification marker in humans, studies addressing the role of genetic changes in apoM and its association with increased risk of diabetes were performed. Hypothetically, SNPs in the human apoM gene that reduce the apoM levels, and resultantly, the S1P levels, could lead to a permanent reduction in S1P-signaling and diabetes risk.

A pioneering study based on the mapping of proteins and genes encoded in the human chromosome 6 in the major histocompatibility complex region suggested a genetic association between apoM and diabetes (53). The study identified a link between apoM and the inflammatory marker AIF1 in patients with T1D. Several studies have investigated multiple SNP variants in the human *APOM* transcript. A study conducted in Peking Union Medical College, Beijing, China and Karolinska Institute, Stockholm, Sweden, included 493 Han Chinese (177 patients with T1D/316 controls) and 225 Swedish (124 patients with T1D/101 controls) individuals (54). The SNP rs805296 was strongly associated with T1D in both the Han Chinese (OR=2.188, CI 95%=1.338–3.581, P=0.002) and Swedish (OR=2.865, CI 95%=1.128–7.278, P=0.021) populations. The study did not report any adjustments for covariables or multiple tests.

More studies have followed, but mostly with a focus on T2D (**Table 2**). One study included 681 patients with T2D and matched controls (57). The rs805296 allele (adjusted OR [95% CI]=1.29 [1.10–1.66], P<0.001) or the C724-del allele (adjusted OR [95% CI]=1.66 [1.40–2.06], P<0.001) indicated a high risk of T2D. Another study included 259 patients with T2D and 76 healthy controls (58). This study could not confirm a higher risk associated with the rs805296 allele. However, the study reported a higher frequency of the C724-del allele in patients with T2D than in healthy controls (P=0.035). C724-del was not associated with a significant change in the plasma apoM levels. A third study conducted in a cohort of 1234 patients with T2D and 606 control participants focused on the rs707922 allele (55). The study found no association between the expression of rs707922 and T2D susceptibility after appropriate adjustment. A fourth study, performed as a Mendelian randomization experiment, reported that the expression of *APOM* SNP variants can reduce the plasma apoM content; however, the variants are not associated with an increased risk of T2D in the general population (56). Two cohorts representative of the Danish general population, the Copenhagen City Heart Study (CCHS, n=8589) and the Copenhagen General Population Study (CGPS; n=93 857), were included. Observational analyses were performed on a subset of participants from the CCHS with available plasma apoM (n=725), and genetic analyses were performed on complete cohorts (n=102 446). During a median follow-up of 16 years (CCHS) and 8 years (CGPS), 563 and 2132 participants developed T2D, respectively. First, an inverse correlation was observed between plasma apoM and T2D risk in a subset of participants from CCHS (hazard ratio between highest vs. lowest quartile (reference)=0.32; 95% confidence interval=0.1–1.01; P for trend=0.02). Second, genotyping of specific SNPs in *APOM* further revealed 10.8% (P=6.2 × 10^-5^) reduced plasma apoM concentration in participants with variant rs1266078 compared to non-carriers. Third, a meta-analysis on data from 599 451 individuals revealed no association between rs1266078 and T2D risk.

**Table 2:** SNP in the human *APOM* gene and its association with changes in plasma apoM levels and risk of diabetes.

SNP	Study design	Number of participants	Effect on plasma apoM	Risk	Reference
rs707921	Case-control	1234 T2D/606 Control	Unchanged	No association	(55)
rs707922	Case-control	1234 T2D/606 Control	Increased	No association	(55)
	Mendelian randomization	123 Carriers/1525 Wild type	Reduced		(56)
rs805264	Case-control	1234 T2D/606 Control	Unchanged	No association	(55)
rs805296	Case-control	177 T1D/316 Controls	Unknown	Increased risk of T1D	(54)
	Case-control	124 T1D/101 Controls	Unknown	Increased risk of T1D	(54)
	Case-control	681 T2D/690 Controls	Unknown	Increased risk of T2D	(57)
	Case-control	259 T2D/76 Controls	Unchanged	No association	(58)
rs805297	Case-control	177 T1D/316 Controls	Unknown	Increased risk of T1D	(54)
	Case-control	124 T1D/101 Controls	Unknown	No association	(54)
	Case-control	681 T2D/690 Controls	Unknown	No association	(57)
	Case-control	259 T2D/76 Controls	Unchanged	No association	(58)
	Case-control	1234 T2D/606 Control	Unchanged	No association	(55)
	Mendelian randomization	916 Carriers/826 Wild type	Unchanged		(56)
rs1266078	Mendelian randomization	1817 T2D/64601 Controls	Reduced	No association	(56)
	Mendelian randomization	9988 T2D/90432 Controls	Unknown	No association	(56)
	Mendelian randomization	19860 T2D/432404 Controls	Unknown	No association	(56)
rs9404941	Case-control	177 T1D/316 Controls	Unknown	No association	(54)
	Case-control	681 T2D/690 Controls	Unknown	No association	(57)
	Case-control	259 T2D/76 Controls	Reduced	No association	(58)
	Case-control	1234 T2D/606 Control	Unchanged	No association	(55)
	Mendelian randomization	163 Carriers/1576 Wild type	Unchanged		(56)
rs150345955	Mendelian randomization	4 Carriers/1557 Wild type	Unchanged		(56)
rs150863040	Mendelian randomization	5 Carriers/1736 Wild type	Unchanged		(56)
C724-del	Case-control	681 T2D/690 Controls	Unknown	Increased risk of T2D	(57)
	Case-control	259 T2D/76 Controls	Unchanged	Increased risk of T2D	(58)

Therefore, the data available from the genetic studies discussed do not indicate that genetic changes in the *APOM* transcript are associated with an increased risk of diabetes in the general population. However, the variants explored thus far are promoter variants of *APOM*, and further studies on coding region variants of *APOM* or the S1P receptor gene may lead to different conclusions.

## Conclusions

Several important aspects of apoM and S1P biology and functionality are largely unknown. Hopefully, it will be confirmed in future studies if apoM, S1P, and/or their complex play potential roles in the development of diabetes or as a target in the treatment of either diabetes or the multiple associated conditions. As a biomarker for diabetes, it is likely that plasma apoM can be used to distinguish between patients with MODY3 and T1D but not T2D. As a biomarker, apoM may also have the potential to predict the risk of mortality in patients with or without diabetes. It remains controversial whether plasma apoM can predict the risk of DN. Clearly, more studies are needed to improve our understanding of these aspects of apoM biology. In such studies, it would be important to remember that hyperinsulinemia exerts opposing effects on plasma apoM and S1P levels, which adds to the complexity of their relationship. A second point of interest is whether apoM/S1P plays a role in the development of T2D. In subsequent studies, the potential bias introduced by the hyperinsulinemia-induced reduction in plasma apoM and increase in plasma S1P levels should be considered carefully. In contrast, the role of the S1P-S1P1 system as an important modulator of the immune response during the development of diabetes seems well established. Further evaluation of immune response-modulating drugs targeting S1P1 will be important and of significant value. Drugs targeting S1P receptors and the artificial apoM/S1P complex have also been developed. However, the use of the apoM/S1P complex in treatment will require an extension of existing knowledge on whether apoM is suitable or unsuitable for lipid, glucose, and insulin metabolism. Lastly, as indicated by the evaluation of the genetic studies published, the hypothesis that a reduction in plasma apoM expression at the gene level is associated with an increased risk of diabetes is yet to be confirmed.

## Author Contributions

The author confirms being the sole contributor of this work and has approved it for publication.

## Funding

This work was funded by the Novo Nordisk Foundation and Augustinus Foundation.

## Conflict of Interest

The author declares that the research was conducted in the absence of any commercial or financial relationships that could be construed as a potential conflict of interest.
